# Prognostic significance of angiogenesis in human pancreatic cancer

**DOI:** 10.1038/sj.bjc.6690248

**Published:** 1999-03

**Authors:** N Ikeda, M Adachi, T Taki, C Huang, H Hashida, A Takabayashi, M Sho, Y Nakajima, H Kanehiro, M Hisanaga, H Nakano, M Miyake

**Affiliations:** Department of Oncology and Department of Thoracic Surgery, Kitano Hospital, Tazuke Kofukai Medical Research Institute,13-3 Kamiyama-cho, Osaka,Kita-ku, 530, Japan; Department of Surgery, Kitano Hospital, Tazuke Kofukai Medical Research Institute,13-3 Kamiyama-cho, Osaka, Kita-ku, 530, Japan; First Department of Surgery, Nara Medical University, 840 Shi Jo-cho,Kashihara-city, Nara, 634, Japan

**Keywords:** PD-ECGF, VEGF, pancreatic cancer, prognosis and intratumoral microvessel density (IMD)

## Abstract

To evaluate whether angiogenic factors are of clinical relevance to actual human pancreatic cancers, we studied the intratumoral microvessel density (IMD), and PD-ECGF, VEGF protein expression in 40 pancreatic cancers using immunohistochemistry. We also investigated *PD-ECGF* and *VEGF* gene expression using reverse transcriptase-PCR (RT-PCR). Of the 40 pancreatic cancers studied, 30 carcinomas (75.0%) were evaluated to be *PD-ECGF*-positive and 10 carcinomas (25.0%) were determined to be *PD-ECGF*-negative. In contrast, 27 carcinomas (67.5%) were evaluated to be *VEGF*-positive, whereas 13 carcinomas (32.5%) were *VEGF*-negative. *VEGF* gene expression was moderately associated with an increase in the IMD (*r*^2^ = 0.181, *P* = 0.006), but no significant relationship was found between *PD-ECGF* gene expression and the IMD (*r*^2^ = 0.093, *P* = 0.059). However, tumours with positive expression for both *PD-ECGF* and *VEGF* had a higher IMD (*P* = 0.027). The results of the immunohistochemistry agreed well with the results of the quantitative RT-PCR. The median survival time of the hypervascular group was significantly shorter than that of the hypovascular group (*P* < 0.0001). In comparing the survival according to *PD-ECGF* and *VEGF* gene expression, the median survival time of the patients with positive *PD-ECGF* expression was significantly shorter than those with negative *PD-ECGF* expression (*P* = 0.040). Furthermore, the median survival time of the patients with positive *VEGF* expression was significantly shorter than those with negative *VEGF* expression (*P* = 0.048). However, the Cox multivariate analysis indicated that the IMD and *VEGF* expression were independent prognostic factors of the various clinicopathologic variables in pancreatic cancer patients (*P* = 0.0021 and *P* = 0.0443, respectively). © 1999 Cancer Research Campaign


British Journal of Cancer (1999) **79**(9/10), 1553–1563
© 1999 Cancer Research Campaign
Article no. bjoc.1998.0248

### Prognostic significance of angiogenesis in human pancreatic cancer

**N Ikeda[1,3], M Adachi[1], T Taki[1], C Huang[1], H Hashida[1], A Takabayashi[2], M Sho[3], Y Nakajima[3], H Kanehiro[3], M Hisanaga[3], H Nakano[3] and M Miyake[1]**

[1]Department V of Oncology and Department of Thoracic Surgery, [2]Department of Surgery, Kitano Hospital, Tazuke Kofukai Medical Research Institute, 13-3 Kamiyama-cho, Kita-ku, Osaka 530, Japan; and [3]First Department of Surgery, Nara Medical University, 840 Shi Jo-cho, Kashihara-city, Nara 634, Japan

**Summary** To evaluate whether angiogenic factors are of clinical relevance to actual human pancreatic cancers, we studied the intratumoral microvessel density (IMD), and PD-ECGF, VEGF protein expression in 40 pancreatic cancers using immunohistochemistry. We also investigated *PD-ECGF* and *VEGF* gene expression using reverse transcriptase-PCR (RT-PCR). Of the 40 pancreatic cancers studied, 30 carcinomas (75.0%) were evaluated to be *PD-ECGF*-positive and 10 carcinomas (25.0%) were determined to be *PD-ECGF*-negative. In contrast, 27 carcinomas (67.5%) were evaluated to be *VEGF*-positive, whereas 13 carcinomas (32.5%) were *VEGF*-negative. *VEGF* gene expression was moderately associated with an increase in the IMD ($r^2 = 0.181$, $P = 0.006$), but no significant relationship was found between *PD-ECGF* gene expression and the IMD ($r^2 = 0.093$, $P = 0.059$). However, tumours with positive expression for both *PD-ECGF* and *VEGF* had a higher IMD ($P = 0.027$). The results of the immunohistochemistry agreed well with the results of the quantitative RT-PCR. The median survival time of the hypervascular group was significantly shorter than that of the hypovascular group ($P < 0.0001$). In comparing the survival according to *PD-ECGF* and *VEGF* gene expression, the median survival time of the patients with positive *PD-ECGF* expression was significantly shorter than those with negative *PD-ECGF* expression ($P = 0.040$). Furthermore, the median survival time of the patients with positive *VEGF* expression was significantly shorter than those with negative *VEGF* expression ($P = 0.048$). However, the Cox multivariate analysis indicated that the IMD and *VEGF* expression were independent prognostic factors of the various clinicopathologic variables in pancreatic cancer patients ($P = 0.0021$ and $P = 0.0443$, respectively).

**Keywords:** PD-ECGF; VEGF; pancreatic cancer; prognosis and intratumoral microvessel density (IMD)

Pancreatic cancer can entail the substantial development of new blood vessels within the tumour tissue, and it is known that the growth and progression of solid tumours depend on such angiogenesis (Folkman, 1995*a*). Angiogenesis is an integral part of the cascade of biologic events involved in tumour metastasis (McCulloch et al, 1995). The mechanisms by which neovascularization stimulates tumour progression are the delivery of the nutrients and oxygen necessary for tumour cell growth, the facilitation of the penetration of tumour cells through the vessel walls and their transport to distant organs, and the secretion of selective cytokines and growth factors from endothelial cells that directly stimulate tumour cells (Fidler and Ellis, 1994). The conversion of tumour cells to an angiogenic phenotype may be preceded by a change in the balance of angiogenic growth factors and angiogenesis inhibitors. To date, many angiogenic factors for pancreatic cancers have been reported, such as transforming growth factor-α (TGF-α) (Barton et al, 1991), transforming growth factor-β (TGF-β) (Friess et al, 1993), acidic fibroblast growth factor (aFGF), basic fibroblast growth factor (bFGF) (Yamanaka et al, 1993), angiogenin (Shimoyama et al, 1996), vascular endothelial growth factor (VEGF) (Itakura et al, 1997) and platelet-derived endothelial cell growth factor (PD-ECGF) (Minari et al, 1997). However, since angiogenesis is composed of multistep processes controlled by various factors, the role of angiogenesis in pancreatic cancer has not been fully elucidated. This study was conducted to determine whether the levels of *PD-ECGF* and *VEGF* gene expression measured in primary tumours from patients with pancreatic cancers were associated with known prognostic factors and patient survival. Furthermore, to clarify whether *PD-ECGF* and *VEGF* correlate with tumour angiogenesis, we examined the intratumoral microvessel density (IMD) by immunohistochemical analysis using anti-CD34 monoclonal antibodies.

*Received 14 April 1998*
*Revised 4 September 1998*
*Accepted 29 September 1998*

*Correspondence to:* M Miyake

### MATERIALS AND METHODS

### Cell lines and tumour tissues

Four human pancreatic cancer cell lines (Capan-2, HPAF-II, PANC-1 and MIA PaCa-2) were maintained in DMEM supplemented with 10% fetal calf serum. Forty patients with pancreatic cancers underwent surgery at the First Department of Surgery, Nara Medical University, between December 1992 and September 1997. The median age of the patients was 63 years, with a range of 47–80 years. When distant metastasis was solitary and resectable, pancreatectomy was performed. Thus, six patients with distant metastasis underwent surgery. Following pancreatectomy, all patients received intraoperative radiation therapy (IORT) with 20 gray (Gy) of electron beam on the retroperitoneal field, including the origins of the portal vein, celiac and superior mesenteric arteries. Moreover,

**Table 1** Relationship between *PD-ECGF* or *VEGF* gene expression, the IMD and known prognostic factors

| Characteristics | Total (n) | PD-ECGF | | | VEGF | | | IMD | | |
|---|---|---|---|---|---|---|---|---|---|---|
| | | (+) | (–) | P-value | (+) | (–) | P-value | (+) | (–) | P-value |
| Age at surgery | | | | | | | | | | |
| ≤ 60 | 27 | 23 | 4 | NS[a,b] | 19 | 8 | NS[a,b] | 12 | 15 | NS[a,b] |
| > 60 | 13 | 7 | 6 | | 8 | 5 | | 7 | 6 | |
| Gender | | | | | | | | | | |
| Male | 30 | 22 | 8 | NS[a,b] | 19 | 11 | NS[a,b] | 12 | 18 | NS[a,b] |
| Female | 10 | 8 | 2 | | 8 | 2 | | 7 | 3 | |
| Tumour status | | | | | | | | | | |
| T1 | 4 | 1 | 3 | 0.0353[c] | 2 | 2 | NS[a,b] | 1 | 3 | 0.0248[c] |
| T2 | 2 | 1 | 1 | | 2 | 0 | | 0 | 2 | |
| T3 | 9 | 6 | 3 | | 6 | 3 | | 8 | 1 | |
| T4 | 25 | 22 | 3 | | 17 | 8 | | 10 | 15 | |
| Nodal status | | | | | | | | | | |
| N0 | 20 | 14 | 6 | NS[a,b] | 12 | 8 | NS[a,b] | 8 | 12 | NS[a,b] |
| N1 | 20 | 16 | 4 | | 15 | 5 | | 11 | 9 | |
| Metastatic status | | | | | | | | | | |
| M0 | 34 | 25 | 9 | NS[a,b] | 22 | 12 | NS[a,b] | 15 | 19 | NS[a,b] |
| M1 | 6 | 5 | 1 | | 5 | 1 | | 4 | 2 | |
| Pathological stage | | | | | | | | | | |
| Stage I | 5 | 1 | 4 | 0.0350[c] | 3 | 2 | NS[a,c] | 1 | 4 | NS[a,c] |
| Stage II | 4 | 3 | 1 | | 3 | 1 | | 4 | 0 | |
| Stage III | 5 | 3 | 2 | | 3 | 2 | | 3 | 2 | |
| Stage IVa | 20 | 18 | 2 | | 13 | 7 | | 7 | 13 | |
| Stage IVb | 6 | 5 | 1 | | 5 | 1 | | 4 | 2 | |
| Histopathological grading | | | | | | | | | | |
| G 1 | 14 | 8 | 6 | 0.0374[c] | 5 | 9 | 0.0058[c] | 4 | 10 | 0.0423[c] |
| G 2 | 21 | 17 | 4 | | 18 | 3 | | 11 | 10 | |
| G 3 | 5 | 5 | 0 | | 4 | 1 | | 4 | 1 | |
| Total number of patients | 40 | 30 | 10 | | 27 | 13 | | 19 | 21 | |

[a]NS, not significant. [b]$\chi^2$ test. [c]Mann–Whitney's *U*-test.

6 mg/m² mitomycin C and 500 mg/m² 5-fluorouracil were administered into the portal vein. Post-operative systemic chemotherapy was not given before recurrence.

All of the tissues were obtained from resected specimens, and then were quickly stored at –80°C until used. The residual specimens were fixed in 10% phosphate buffered formalin and embedded in paraffin. In order to ascertain the presence of cancer cells, one half of each fresh tumour tissue specimen was immediately embedded in optimum cutting temperature (OCT)-compound (Miles, Kankakee, IL, USA), and frozen sections were then cut on the cryostat to a thickness of 6 µm and immediately we reviewed the H&E-stained slides of the tumour specimens. After the connective tissues were trimmed off, the other half of the tumour specimen containing greater than 80% cancer cells of all tissue cells was selected for the RT-PCR analysis. The tumours were classified according to the tumour–node–metastasis (TNM) staging system (Sobin and Wittekind, 1997). The clinical characteristics of these patients are presented in Table 1. The median follow-up for all patients was 25.9 months, with a range of 5–62 months.

### RT-PCR analysis

The total cellular RNA was extracted from the cell lines and frozen tumour tissues by the acid guanidinium thiocyanate procedure (Chomczynski and Sacchi, 1987). In a preliminary study, we confirmed that the human colon cancer cell line colo 201 had *PD-ECGF* and *VEGF* mRNA expression as a positive control. First-strand cDNA synthesis was performed with 5 µg of total RNA, using a cDNA synthesis kit (Pharmacia, Piscataway, NJ, USA) and following the manufacturer's protocol. For PCR amplification, we used a 1-µl aliquot of the reaction mixture. To obtain reproducible quantitative performance of RT-PCR assays for *PD-ECGF* and *VEGF*, we titrated the amount of starting cDNA against the number of amplification cycles. The generated cDNAs were amplified using primers for *PD-ECGF* (5′-CTGATCCGCAT-GAAGCGAGAC-3′ and 5′-CTCCACGAGTTTCTTACTGAGA-AT-3′) (Ishikawa et al, 1989), *VEGF* (5′-GAAGTGGTGAAGTT-CATGGATGTC-3′ and 5′-CGATCGTTCTGTATCAGTCTTTCC-3′) (Tischer et al, 1991), and *β-actin* (5′-GAGAGATGACCCA-GATCATGT-3′ and 5′-ACTCCATGCCCAGG AAGGAAGG-3′) (Nakajima-Iijima et al, 1985). All subsequent assays were carried out using those parameters that yielded an amplification of both *PD-ECGF*, *VEGF* and *β-actin* (the internal control) within a linear range. The *PD-ECGF* and *VEGF* reaction mixture was subjected to 28 PCR amplification cycles of 40 s, each at 94°C, 40 s at 60°C, and 90 s at 72°C. The same PCR conditions of 20 cycles were used to amplify the *β-actin* DNA. Tubes containing all of the ingredients except for the templates were included in all runs, and served

© Cancer Research Campaign 1999

as negative reaction controls. The amplified DNA samples were run on a 1% agarose gel, and the bands were visualized with ethidium bromide and photographed with a Polaroid camera. Densitometric analysis of the photographic negatives was used for band quantification.

### Specimen classification based on RT-PCR results

The densitometric values obtained for the *PD-ECGF* and *VEGF* bands in a given tumour tissue sample were divided by the corresponding value for *β-actin*, and the ratio was referred to as the gene expression ratio for each gene. VEGF has four isoforms (121, 165, 189 and 206 amino acids), resulting from the alternative splicing of the primary transcript (Tischer et al, 1991); therefore, we divided the value obtained for *VEGF*121 by the corresponding value for *β-actin*. The expression ratio in a given tumour was then divided by the expression ratio of the human colon cancer cell line colo 201 in order to obtain the gene conservation rates for *PD-ECGF* and *VEGF*. Since most prognostic factors are usually considered as dichotomized, discontinuous variables, a cut-off point was selected to give the optimal separation between a low and high risk of the overall survival as described previously (Tandon et al, 1990). Thus, the most significant *P*-value for the survival was found by a relative cut-off point for positive PD-ECGF expression of 1.5. When the conservation rate value of a given specimen was > 1.5, it was considered to indicate positive *PD-ECGF* gene expression. If the value was ≤ 1.5, it was considered to represent negative expression. This was true of a relative cut-off point for positive VEGF expression of 1.0. When the conservation rate value of a given specimen was > 1.0, it was considered to indicate positive *VEGF* gene expression, whereas a ratio ≤ 1.0 was considered to represent negative expression.

### Immunohistochemical staining of PD-ECGF, VEGF and CD34

We used a mouse monoclonal antibody obtained from Nippon Roche Research Center at a 1:100 dilution for PD-ECGF, a rabbit polyclonal antibody (Santa Cruz Biotechnology, Santa Cruz, CA, USA) at a 1:200 dilution for VEGF, and a mouse monoclonal antibody (Nichirei Corporation, Tokyo, Japan) at a 1:20 dilution for CD34. After the sections were incubated with normal goat serum to block the non-specific binding, they were treated with the anti-PD-ECGF, anti-CD34 or anti-VEGF antibodies at room temperature. Immunostaining was performed by the streptavidin-biotin (SAB)s method using Histofine SAB-PO (M) and SAB-PO (R) kits (Nichirei, Tokyo, Japan). The staining was visualized with 3,3′-diaminobenzidine tetrahydrochloride. The slides were counterstained with haematoxylin. Sections incubated with mouse myeloma $SP_2$ supernatant or normal rabbit IgG served as negative reaction controls.

### Evaluation of immunostaining and microvessel counting

At least 200 tumour cells were scored per 400 × field. Positive tumour cells were stained to an intensity equivalent to normal fibroblasts. When > 10% of the carcinoma cells in a given specimen were positively stained, the sample was classified as PD-ECGF-positive (+) or VEGF-positive (+), and when ≤ 10% of the cells were stained, the sample was designated as negative (–). For the microvessel counting, the four most highly vascularized areas were counted in 200 × fields (0.785 $mm^2$ per field), and the average counts were recorded. The mean microvessel count of these tumours was 58.6 ± 17.4. Therefore, we classified the tumours into two groups: tumours with a microvessel count >58.6 were classified as hypervascular, and tumours with a count ≤ 58.6 were classified as hypovascular. The evaluation of PD-ECGF and VEGF staining and the microvessel counting were performed by two investigators who had no knowledge of the patients' clinicopathologic factors or clinical outcomes.

### Statistical analysis

The overall cancer-specific survival was defined from the date of the operation to the data of death due to cancer. The statistical significance of the difference between the incidence of *PD-ECGF* or *VEGF* gene expression and several clinical and pathological parameters was assessed by the $\chi^2$ test or by Mann–Whitney's U-test. Pearson's correlation coefficient was performed to examine correlation between *PD-ECGF* or *VEGF* gene expression and the IMD. The Kaplan–Meier method was used to estimate the probability of overall survival as a function of time (Kaplan and Meier, 1958) and was compared using the log-rank test (Mantel, 1966). Multivariate analysis were performed by using the Cox regression model (Cox 1972) with the SAS statistical package (SAS Institute, Cary, NC, USA), and nine factors (*PD-ECGF* status, *VEGF* status, intratumoral microvessel density (IMD), histopathological grading, tumour status, nodal status, metastatic status, gender and age at surgery) were studied; scores were also assigned to each variable for the regression analysis. All *P*-values were based on a two-tailed statistical analysis and a $P < 0.05$ was considered to indicate statistical significance.

### RESULTS

### *PD-ECGF* and *VEGF* gene expression in pancreatic cancer cell lines and tumour tissues analysed by RT-PCR

*PD-ECGF* mRNA was detected in three (75.0%) of the four human pancreatic cancer cell lines by the RT-PCR assay; only the MIA PaCa-2 cell line had no *PD-ECGF* gene expression (Figure 1A). Of the 40 pancreatic cancers studied, 30 carcinomas (75.0%) were evaluated to be *PD-ECGF*-positive, and 10 carcinomas (25.0%) were determined to be *PD-ECGF*-negative (Figure 1A). The *PD-ECGF* gene expression rate ranged from 0 to 6.34, with a mean value of 2.71. In contrast, all four human pancreatic cancer cell lines had *VEGF* mRNA expression (Figure 1B). Twenty-seven carcinomas (67.5%) were evaluated to be *VEGF*-positive, and 13 carcinomas (32.5%) were *VEGF*-negative (Figure 1B). The *VEGF* gene expression rate ranged from 0 to 3.24, with a mean value of 1.43. There was no significant correlation between the gene expression ratios of *PD-ECGF* and *VEGF* ($r^2$= 0.025).

### PD-ECGF and VEGF protein expression analysed by immunohistochemistry

The PD-ECGF protein positive ratio obtained by immunohistochemistry was 67.5% (27 out of 40). PD-ECGF protein was found mainly in the cytoplasm and nuclei of the cancer cells. In addition,

© Cancer Research Campaign 1999 

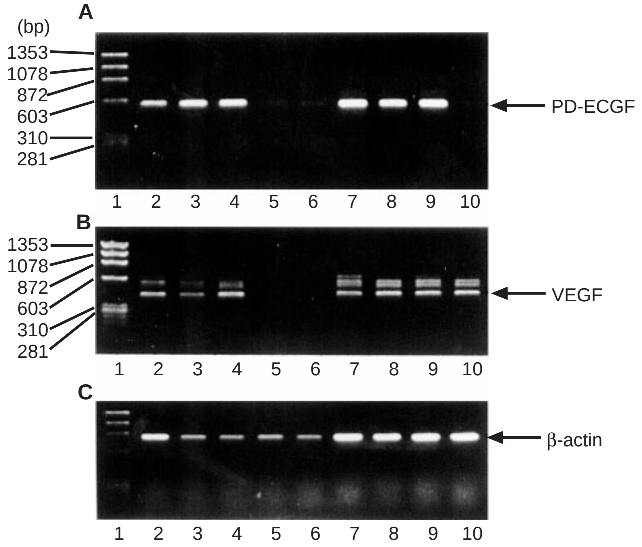

**Figure 1** Agarose gel electrophoresis of RT-PCR amplified PD-ECGF cDNA (**A**). Lane 1, size marker; lane 2, human colon cancer cell line colo 201 (positive control); lanes 3–4, primary pancreatic adenocarcinomas evaluated as *PD-ECGF* gene expression positive; lanes 5–6, primary pancreatic adenocarcinomas evaluated as *PD-ECGF* gene expression negative; lanes 7–10, human pancreatic cancer cell lines (Capan-2, HPAF-II, PANC-1 and MIA PaCa-2). Agarose gel electrophoresis of RT-PCR amplified VEGF cDNA (**B**). Lane 1, size marker; lane 2, human colon cancer cell line colo 201 (positive control); lanes 3–4, primary pancreatic adenocarcinomas evaluated as *VEGF* gene expression positive; lanes 5–6, primary pancreatic adenocarcinomas evaluated as *VEGF* gene expression negative; lanes 7–10, human pancreatic cancer cell lines (Capan-2, HPAF-II, PANC-1 and MIA PaCa-2). Agarose gel electrophoresis of RT-PCR amplified β-actin cDNA (**C**)

the islets, macrophages and fibroblasts were also stained (Figure 2A). The *PD-ECGF* gene expression evaluated by the RT-PCR was highly associated with *PD-ECGF* protein expression by the immunohistochemical staining ($r^2 = 0.51$, $P < 0.0001$) (Figure 3A). Overall, the results of the PD-ECGF immunohistochemistry agreed well with the RT-PCR assay data, and 90.0% of the samples coincided exactly. On the other hand, the VEGF protein positive ratio was 60.0% (24 out of 40). VEGF protein was found mainly in the cytoplasm and islets. Occasionally, the nuclei of the cancer and stromal cells, which may have been macrophages or fibroblasts, were also stained (Figure 2B). The *VEGF* gene expression evaluated by the RT-PCR was also associated with VEGF protein expression by the immunohistochemical staining ($r^2 = 0.52$, $P < 0.0001$) (Figure 3B). These results also agreed well with the RT-PCR assay data, and 92.5% of the samples coincided exactly. In the case of a discrepancy, the results from the RT-PCR analysis were used in the specimen classification.

### Microvessel staining

A single microvessel was defined as any brown immunostained endothelial cells separated from the adjacent microvessels, tumour cells, and other connective tissue elements (Figure 2C). The microvessel count for the 40 tumours ranged from 33.0 to 117.0, with a mean microvessel count of 58.6 ± 17.4. The 40 patients were then classified into two groups: 19 patients with hypervascular tumours and 21 patients with hypovascular tumours.

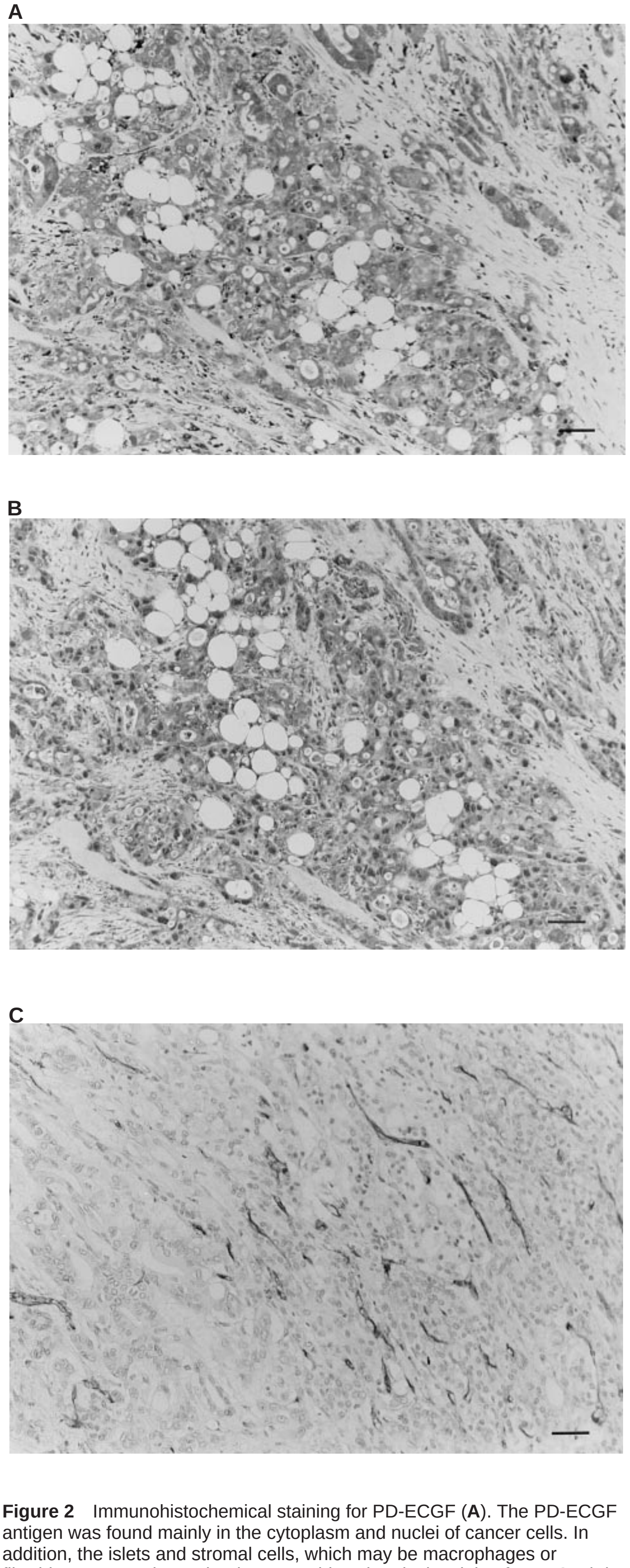

**Figure 2** Immunohistochemical staining for PD-ECGF (**A**). The PD-ECGF antigen was found mainly in the cytoplasm and nuclei of cancer cells. In addition, the islets and stromal cells, which may be macrophages or fibroblasts, were also stained. Immunohistochemical staining for VEGF (**B**). The VEGF antigen was found mainly in the cytoplasm and nuclei of cancer cells. Occasionally, the nuclei of cancer and stromal cells, which may be macrophages or fibroblasts, were also stained. Immunohistochemical staining for microvessel (**C**). A single microvessel was defined as any brown immunostained endothelial cell separated from the adjacent microvessels, tumour cells, and other connective tissue elements. **A** and **B**: original magnification × 100. Bars = 20 μm. **C**: original magnification × 200. Bars = 10 μm

© Cancer Research Campaign 1999

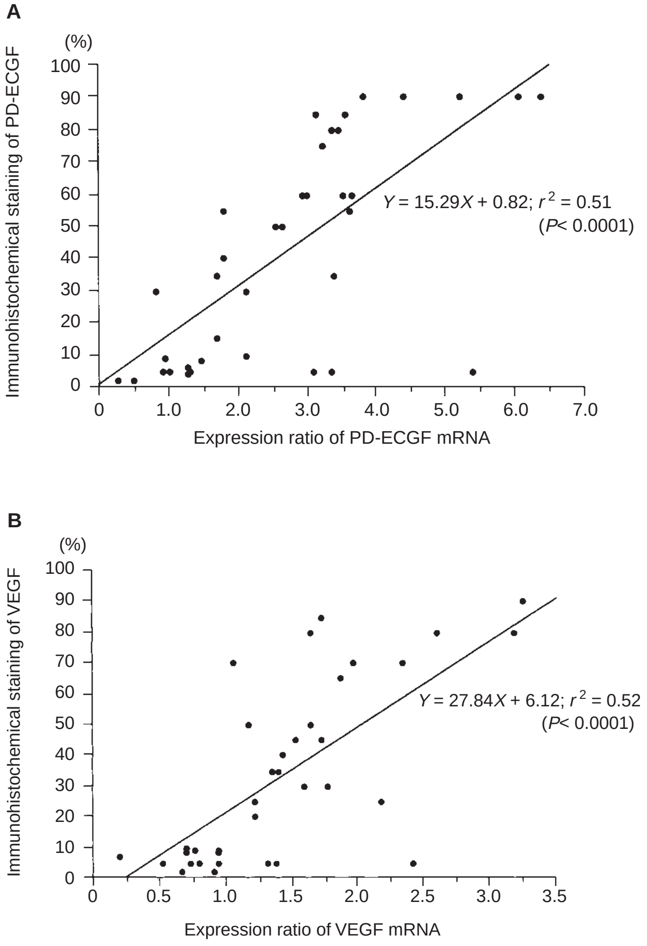

**Figure 3** Pearson's correlation coefficient between gene conservation rate by RT-PCR and protein expression by immunohistochemistry. The association between *PD-ECGF* gene expression and PD-ECGF protein expression (**A**). The association between *VEGF* gene expression and VEGF protein expression (**B**)

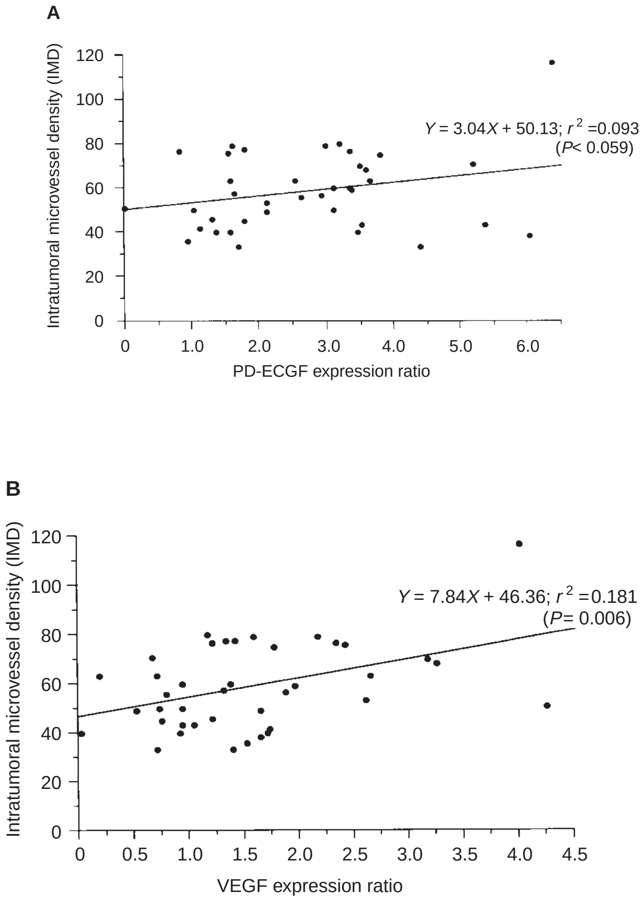

**Figure 4** There was no significant correlation between the expression ratio of *PD-ECGF* and the IMD (**A**). However, the expression ratio of *VEGF* was moderately associated with an increase in the intratumoral microvessel density (IMD) (**B**)

### Relationships between *PD-ECGF* or *VEGF* gene expression and the IMD

No significant relationship was found between the expression ratio of *PD-ECGF* and the IMD (Figure 4A; $r^2 = 0.093$, $P = 0.059$), but the expression ratio of *VEGF* was moderately associated with an increase in the IMD (Figure 4B; $r^2 = 0.181$, $P= 0.006$).

### Relationship between PD-ECGF or VEGF gene expression, the IMD and known prognostic factors

The relationship between *PD-ECGF* gene expression and various prognostic factors is shown in Table 1. *PD-ECGF* gene expression was associated with the histopathological grading ($P = 0.0374$), the tumour status ($P = 0.0353$), and the pathological stage ($P = 0.0350$). 100% of the G3 grade patients had positive gene expression, as compared with 57.1% of the G1 grade patients. With respect to the tumour status, the proportion of patients whose tumours had positive gene expression increased from 25.0% of those with a T1 status to 88.0% of those patients with a T4 status. In addition, 83.3% of the Stage IVb patients had positive gene expression compared with 22.0% of the Stage I patients. In contrast, there was no significant relationship between *PD-ECGF* gene expression and the patients' age at surgery, gender, nodal status or metastatic status.

The relationship between *VEGF* gene expression and various prognostic factors is shown in Table 1. *VEGF* gene expression was also significantly associated with the histopathological grading ($P = 0.0058$); 80.0% of the G3 grade patients had positive gene expression as compared with 35.7% of the G1 grade patients. In contrast, there was no statistically significant relationship between *VEGF* gene expression and the other factors.

The IMD was significantly associated with the histopathological grading ($P = 0.0423$) and tumour status ($P = 0.0248$); 80.0% of the G3 grade patients were classified to the hypervascular group, as compared with 28.6% of the G1 grade patients. With respect to the tumour status, the proportion of patients with hypervascular tumours increased from 25.0% of those with a T1 status to 40.0% of those patients with a T4 status. There was no statistically significant relationship between the IMD and the other factors.

© Cancer Research Campaign 1999

**Table 2** Association of *PD-ECGF* gene expression with the survival of pancreatic cancer patients

| Characteristics | Total (*n*) | Median survival time (month) *PD-ECGF* (+) | Median survival time (month) *PD-ECGF* (–) | Overall survival rate (%) *PD-ECGF* (+) | Overall survival rate (%) *PD-ECGF* (–) | *P*-value |
|---|---|---|---|---|---|---|
| Age at surgery (yr) | | | | | | |
| ≤ 60 | 27 | 7.8 | 6.8 | 0 | 50 | 0.287 |
| > 60 | 13 | 10.6 | NR[a] | 0 | 75 | 0.090 |
| Gender | | | | | | |
| Male | 30 | 8.7 | NR[a] | 0 | 57.1 | 0.104 |
| Female | 10 | 10.6 | NR[a] | 0 | 100 | ND[b] |
| Tumour status | | | | | | |
| T1 | 4 | NR[a] | NR[a] | 100 | 100 | ND[b] |
| T2 | 2 | 11.3 | NR[a] | 0 | 100 | ND[b] |
| T3 | 9 | 4.9 | 6.3 | 0 | 0 | 0.702 |
| T4 | 25 | 9.5 | NR[a] | 0 | 66.7 | 0.594 |
| Nodal status | | | | | | |
| N0 | 20 | 14.0 | NR[a] | 0 | 80 | 0.041 |
| N1 | 20 | 8.7 | 6.8 | 0 | 33.3 | 0.702 |
| Metastatic status | | | | | | |
| M0 | 34 | 11.3 | NR[a] | 0 | 71.4 | 0.035 |
| M1 | 6 | 6.4 | 5.0 | 0 | 0 | 0.351 |
| Pathological stage | | | | | | |
| Stage I | 5 | NR[a] | NR[a] | 100 | 100 | ND[b] |
| Stage II | 4 | 24.3 | 6.3 | 0 | 0 | 0.515 |
| Stage III | 5 | 11.3 | 6.8 | 0 | 0 | 0.515 |
| Stage IVa | 20 | 10.6 | NR[a] | 0 | 100 | 0.351 |
| Stage IVb | 6 | 6.4 | 5.0 | 0 | 0 | 0.351 |
| Histopathological grading | | | | | | |
| G 1 | 14 | 9.5 | NR[a] | 16.7 | 75 | 0.171 |
| G 2 | 21 | 10.6 | 6.3 | 0 | 50 | 0.868 |
| G 3 | 5 | 8.3 | – | 0 | – | – |
| Total number of patients | 40 | 9.5 | NR[a] | 0 | 62.5 | 0.040 |

[a]NR, not reached. [b]ND, not done.

### Association of *PD-ECGF* and *VEGF* gene expression and the IMD with the survival of pancreatic cancer patients

The prognosis of patients with pancreatic cancer was very poor, and the overall survival rates for almost all of the factors were 0%. Of the 40 patients with pancreatic cancer, the median survival time of the patients with positive *PD-ECGF* gene expression was shorter than that of the patients with negative *PD-ECGF* gene expression, as demonstrated in Table 2 (9.5 months vs not reached, $P$ = 0.040; Figure 5A). This difference was particularly evident in those patients with a M0 metastatic status (11.3 months vs not reached; $P$ = 0.035). Furthermore, there was a medical significant difference in N0 nodal status (14.0 months vs not reached; $P$ = 0.041). The median survival time of the patients with positive *VEGF* expression was lower than that of the patients with negative *VEGF* expression, as demonstrated in Table 3 (7.5 months vs 14.0 months; $P$ = 0.048; Figure 5B). This difference was particularly evident in male and elderly patients ($P$ = 0.027 and $P$ = 0.039, respectively).

On the other hand, the median survival time of the hypervascular group was shorter than that of the hypovascular group in Table 4 (6.3 months vs 18.8 months; $P$ < 0.0001; Figure 5C). In addition, there were significant differences noted for the following variables: male (6.3 months vs 21.6 months; $P$ < 0.0001), elderly patients (6.3 months vs 18.8 months; $P$ = 0.0035), T4 tumour status (7.5 months vs 14.0 months; $P$ = 0.0004), N0 or N1 nodal status (4.7 months vs 27.6 months; $P$ = 0.0087 and 6.8 months vs 13.2 months; $P$ = 0.0038), M0 metastatic status (6.3 months vs 21.6 months; $P$ < 0.0001), Stage IVa (6.1 months vs 18.8 months; $P$ < 0.0001) and G1 or G2 histopathological grading (6.8 months vs 14 months; $P$ = 0.0027 and 6.1 months vs 21.6 months; $P$ = 0.0070).

### Prognostic value of *PD-ECGF* status, *VEGF* status, and IMD

We studied the independent prognostic value of each variable used in the Cox regression analysis as shown in Table 5. The IMD and *VEGF* status were found to be significant independent prognostic factors ($P$ = 0.0021 and $P$ = 0.0443, respectively). The other variables (*PD-ECGF* status, histopathological grading, tumour status, nodal status, metastatic status, gender, age at surgery) were not significant.

### DISCUSSION

Tumour growth depends on angiogenesis to a large degree, which means that the tumours are dependent on the ingrowth of a

 *© Cancer Research Campaign 1999*

**Table 3** Association of *VEGF* gene expression with the survival of pancreatic cancer patients

| Characteristics | Total (*n*) | Median survival time (month) | | Overall survival rate(%) | | *P*-value |
|---|---|---|---|---|---|---|
| | | *VEGF* | | *VEGF* | | |
| | | (+) | (–) | (+) | (–) | |
| Age at surgery (yr) | | | | | | |
| ≤ 60 | 27 | 6.1 | 8.7 | 28.6 | 0 | 0.564 |
| > 60 | 13 | 8.3 | NR[a] | 0 | 51.4 | 0.039 |
| Gender | | | | | | |
| Male | 30 | 6.8 | 14.0 | 11.8 | 23.3 | 0.027 |
| Female | 10 | 10.6 | NR[a] | 0 | 100 | ND[b] |
| Tumour status | | | | | | |
| T1 | 4 | NR[a] | NR[a] | 100 | 100 | ND[b] |
| T2 | 2 | 11.3 | – | 50.0 | – | ND[b] |
| T3 | 9 | 5.0 | NR[a] | 0 | 100 | ND[b] |
| T4 | 25 | 8.3 | 13.2 | 0 | 0 | 0.246 |
| Nodal status | | | | | | |
| N0 | 20 | 18.8 | 27.6 | 18.5 | 28.6 | 0.303 |
| N1 | 20 | 7.5 | 13.2 | 0 | 0 | 0.306 |
| Metastatic status | | | | | | |
| M0 | 34 | 10.6 | 27.6 | 9.6 | 25.9 | 0.086 |
| M1 | 6 | 5.0 | 8.7 | 0.0 | 0 | 0.156 |
| Pathological stage | | | | | | |
| Stage I | 5 | NR[a] | NR[a] | 100 | 100 | ND[b] |
| Stage II | 4 | 6.3 | NR[a] | 0 | 100 | ND[b] |
| Stage III | 5 | 6.8 | NR[a] | 0 | 100 | ND[b] |
| Stage IVa | 20 | 10.6 | 13.2 | 0 | 0 | 0.365 |
| Stage IVb | 6 | 5.0 | 8.7 | 0 | 0 | 0.156 |
| Histopathological grading | | | | | | |
| G1 | 14 | 11.3 | 14.0 | 33.3 | 42.9 | 0.579 |
| G2 | 21 | 7.5 | 27.6 | 0 | 0 | 0.078 |
| G3 | 5 | 4.2 | 8.7 | 0 | 0 | 0.754 |
| Total number of patients | 40 | 7.5 | 14.0 | 7.8 | 23.3 | 0.048 |

[a]NR, not reached. [b]ND, not done.

vascular supply from the surrounding tissues in order to proliferate and metastasize (Folkman, 1995*a*; McCulloch et al, 1995). Any individual tumour may have dominant angiogenic factors that favour the imbalance of positive regulators and negative ones to induce angiogenesis. Of these factors, PD-ECGF and VEGF are particularly important angiogenic factors. PD-ECGF is an endothelial cell mitogen of relative molecular mass purified to homogeneity from human platelets, and has chemotactic activity for endothelial cells in vitro and angiogenic activity in vivo (Ishikawa et al, 1989). In 1992, PD-ECGF was identified as being homologous to thymidine phosphorylase (dThdPase), which catalyses the reversible phosphorylation of thymidine to thymine and 2-deoxyribose-1-phosphate (Furukawa et al, 1992; Usuki et al, 1992); this enzymatic activity is crucial for the angiogenic activity. Takahashi et al (1996) have demonstrated that PD-ECGF was expressed in infiltrating cells in most of colon cancers, but rarely in tumour epithelium. However, in our cases three of four pancreatic cell lines have *PD-ECGF* gene expression and the PD-ECGF protein positive ratio obtained by immunohistochemistry was 67.5% (27 out of 40).

On the other hand, VEGF is a potent and widely distributed angiogenic peptide (Dvorak et al, 1995). This growth factor is a dimeric 34–42 kD glycosylated basic protein with moderate affinity for heparin, and is encoded in four molecular isoforms (121, 165, 189 and 206 amino acids) resulting from the alternative splicing of the primary transcript (Tischer et al, 1991). Two smaller isoforms (VEGF121 and 165) are secretary proteins, whereas the other two larger isoforms (VEGF189 and 206) are bound to heparin-containing proteoglycans or the cell surface or basement membrane (Houck et al, 1992). The former isoforms induce their angiogenic effects by binding to the specific transmembrane tyrosine kinase receptors KDR/flk-1 and flt-1, also termed VEGFR-1 and VEGFR-2, respectively, which are selectively expressed on vascular endothelial cells (Dvorak et al, 1995). Aberrant levels of VEGF have also been found in the sera from 10% of the patients with early-stage breast cancer (Yamamoto et al, 1996). However, the biologic significance of circulating endothelial growth factors is presently not known. In this study, since *VEGF*121 and *VEGF*165 mRNAs were potently expressed and high *VEGF*121 expression was found in the tumours with elevated vascularization (Houck et al, 1992), we studied the *VEGF*121 expression. VEGF expression increases in response to several stimuli such as hypoxia (Brogi et al, 1996), certain oncogene products including mutant ras genes (Rak et al, 1995) and the overexpression of transforming growth factor (Detmar et al, 1994). In addition to tumour growth and metastasis, the endogenous upregulation of VEGF has been implicated as the basis for the angiogenesis associated with diabetic retinopathy (Aiello et al, 1994), rheumatoid arthritis (Fava et al, 1994), and wound healing (Tsurumi et al, 1997).

Recently, many studies have suggested that the IMD was a significant predictor of an increased risk of metastasis and a poorer

© Cancer Research Campaign 1999

**Table 4** Association of IMD with the survival of pancreatic cancer patients

| | | Median survival time (month) | | Overall survival rate(%) | | |
|---|---|---|---|---|---|---|
| | | IMD | | IMD | | |
| **Characteristics** | **Total (*n*)** | **(+)** | **(–)** | **(+)** | **(–)** | ***P*-value** |
| Age at surgery (yr) | | | | | | |
| ≤ 60 | 27 | 6.1 | 27.6 | 0 | 30 | 0.0750 |
| > 60 | 13 | 6.3 | 18.8 | 0 | 19.7 | 0.0035 |
| Gender | | | | | | |
| Male | 30 | 6.3 | 21.6 | 0 | 25.2 | < 0.0001 |
| Female | 10 | 6.1 | 18.8 | 0 | 0 | 0.4385 |
| Tumour status | | | | | | |
| T1 | 4 | NR[a] | NR[a] | 100 | 100 | ND[b] |
| T2 | 2 | – | 11.3 | – | 50 | ND[b] |
| T3 | 9 | 6.3 | NR[a] | 0 | 100 | ND[b] |
| T4 | 25 | 7.5 | 14.0 | 0 | 0 | 0.0004 |
| Nodal status | | | | | | |
| N0 | 20 | 4.7 | 27.6 | 0 | 36.6 | 0.0087 |
| N1 | 20 | 6.8 | 13.2 | 0 | 0 | 0.0038 |
| Metastatic status | | | | | | |
| M0 | 34 | 6.3 | 21.6 | 0 | 25.5 | < 0.0001 |
| M1 | 6 | 8.3 | 5.0 | 0 | 0 | 0.3636 |
| Pathological stage | | | | | | |
| Stage I | 5 | NR[a] | NR[a] | 100 | 100 | ND[b] |
| Stage II | 4 | 6.3 | – | 0 | – | ND[b] |
| Stage III | 5 | 4.9 | 11.3 | 0 | 0 | 0.0900 |
| Stage IVa | 20 | 6.1 | 18.8 | 0 | 0 | < 0.0001 |
| Stage IVb | 6 | 8.3 | 5.0 | 0 | 0 | 0.364 |
| Histopathological grading | | | | | | |
| G1 | 14 | 6.8 | 14.0 | 0 | 44.4 | 0.0027 |
| G2 | 21 | 6.1 | 21.6 | 0 | 0 | 0.0070 |
| G3 | 5 | 4.2 | 18.8 | 0 | 0 | 0.1564 |
| Total number of patients | 40 | 6.3 | 18.8 | 0 | 22.9 | < 0.0001 |

[a]NR, not reached. [b]ND, not done.

overall survival of solid tumour (Weidner and Folkman, 1996). Our study also showed that the IMD was the most significant indicator of a poor prognosis among *PD-ECGF* expression, *VEGF* expression and the IMD. In colon cancers, PD-ECGF has been reported to be closely related to the IMD (Takebayashi et al, 1996), and in gastric cancers VEGF was also correlated with the IMD (Takahashi et al, 1998). However, in pancreatic cancers, no significant relationship was found between *PD-ECGF* expression and the IMD ($r^2$ = 0.093, $P$ = 0.059); only *VEGF* expression was moderately correlated with the IMD ($r^2$ = 0.181, $P$ = 0.006). These data agreed well with the previous report by Itakura et al (1997). Indeed, *PD-ECGF* gene expression correlated with a poor prognosis in patients with pancreatic cancer, but these findings suggest that *PD-ECGF* may not play a more important role in angiogenesis than *VEGF*. However, tumours with both positive *PD-ECGF* and *VEGF* gene expression had a higher IMD than those with either negative or both negative expression ($P$ = 0.027; $\chi^2$ test). Furthermore, these patients with both positive *PD-ECGF* and *VEGF* gene expression had a significantly poorer prognosis than those with either negative or both negative expression ($P$ = 0.010). The activity of one factor may therefore facilitate the angiogenic activity of the other factor. For example, the chemotactic activity of PD-ECGF might facilitate tubule formation after the proliferation of the endothelial cells secondary to VEGF activity (Takahashi et al, 1998). However, apart from PD-ECGF and VEGF, other dominant angiogenic factors that might directly correlate with angiogenesis in pancreatic cancers should be considered. Furthermore, the coexpression of diverse endothelial growth factors may have a synergistic effect on angiogenesis and ultimately, neovascularization is probably the result of the net local balance between angiogenic stimuli and angioinhibitory pathways (Folkman, 1995*a*; Hanahan and Folkman, 1996). Therefore, the IMD may provide prognostic information on the clinical behaviour of pancreatic cancers.

We found that an assessment of the *PD-ECGF* and *VEGF* gene expression levels by RT-PCR was useful. In general, immunohistochemical methods are evaluated by subjective criteria. Therefore, we believe that the RT-PCR assay we used is a valuable and objective method of detecting PD-ECGF and VEGF. However, a histologic control is very important for evaluating blind RNA analysis, so we initially selected specimens containing greater than 80% cancer cells of all tissue cells selected for the RT-PCR analysis and these results were ascertained by immunohistochemistry. In addition, the potential advantages of this assay system are that it can distinguish the different isoforms of VEGF and that it may lead to the identification of the cellular sources of

 © Cancer Research Campaign 1999

**Table 5** Multivariate Cox analysis of overall survival of 40 patients with pancreatic cancer

| Variable | Assigned score | β | S.E. | χ² | Hazard ratio (95% CI) | *P*-value |
|---|---|---|---|---|---|---|
| IMD | | | | | | |
| Hypervascular | 0 | – 2.07 | 0.672 | 9.499 | 0.126 (0.034–0.471) | 0.0021 |
| Hypovascular | 1 | | | | | |
| PD-ECGF | | | | | | |
| Positive | 0 | – 1.11 | 0.983 | 1.276 | 0.329 (0.048–2.262) | 0.2586 |
| Negative | 1 | | | | | |
| VEGF | | | | | | |
| Positive | 0 | – 1.747 | 0.868 | 4.047 | 0.174 (0.032–0.956) | 0.0443 |
| Negative | 1 | | | | | |
| Histopathological grading | | | | | | |
| G1 | 1 | – 0.856 | 0.688 | 1.549 | 0.425 (0.110–1.636) | 0.2133 |
| G2 | 2 | | | | | |
| G3 | 3 | | | | | |
| Tumour status | | | | | | |
| T1 | 1 | 1.046 | 0.599 | 3.052 | 2.846(0.880–9.200) | 0.0806 |
| T2 | 2 | | | | | |
| T3 | 3 | | | | | |
| T4 | 4 | | | | | |
| Nodal status | | | | | | |
| N0 | 0 | – 0.174 | 0.607 | 0.082 | 0.840 (0.256–2.759) | 0.7740 |
| N1 | 1 | | | | | |
| Metastatic status | | | | | | |
| M0 | 0 | 0.788 | 0.865 | 0.83 | 2.198 (0.404–11.976) | 0.3624 |
| M1 | 1 | | | | | |
| Gender | | | | | | |
| Male | 0 | 1.346 | 0.732 | 3.378 | 3.841 (0.914–16.139) | 0.0661 |
| Female | 1 | | | | | |
| Age | | | | | | |
| ≤ 60 | 0 | – 0.629 | 0.655 | 0.922 | 0.533 (0.148–1.926) | 0.3369 |
| > 60 | 1 | | | | | |

VEGF. In fact, some stromal cells have also been shown to be able to produce this endothelial growth factor (Yoshiji et al, 1996).

Thus far, the angiogenic properties of pancreatic cancers have been unclear because pancreatic cancers have been known to be hypovascular tumours via roentgenographic imaging. In this study, we demonstrated that angiogenesis depended on VEGF more than PD-ECGF, and the tumours with both *PD-ECGF* and *VEGF* gene expression had a higher IMD ($P = 0.027$; $\chi^2$ test). The results of our study suggested that VEGF was the main angiogenic pathway in human pancreatic cancers. Another important clinical implication of our results, beyond the prognosis, is that VEGF may be a potentially useful target for the pharmacologic inhibition of angiogenesis (Folkman, 1995*a*; Folkman, 1995*b*). Some investigations have succeeded in the suppression of tumour growth in vivo through the inhibition of angiogenesis by blocking the biologic functions of VEGF. For example, neutralizing antibodies against VEGF (Kim et al, 1993), cytotoxic conjugates of recombinant VEGF with diphtheria toxin (Ramakrishnan et al, 1996), and genetic approaches using retroviruses encoding dominant negative mutant KDR/flk-1 receptors capable of infecting and inhibiting the growth of target endothelial cells (Millauer et al, 1994) have all brought about angiogenesis-related tumour regression in animal models. Human pancreatic cancer still has a very poor prognosis even after a curative resection (Nitecki et al, 1995). Only 8% of the patients are resectable at the time of diagnosis, and the overall 5-year survival rate is less than 10% because most patients die from recurrent diseases (Bramhall et al, 1995; Gastrointestinal Tumor Study Group, 1987). Based on the high rate of recurrence, surgery alone seems to be inadequate for the treatment of patients with pancreatic cancer. Indeed, new clinical approaches such as, for example adjuvant chemotherapy plus radiation therapy (chemoradiation) after surgery or before surgery have been attempted and only a little improvement was observed in the survival rates of patients with pancreatic cancer, but the prognosis has remained worse than other cancers, such as breast cancer, colon cancer and gastric cancer (Ozaki et al, 1996; Schnall et al, 1996; Kamthan et al, 1997). In addition, most patients with pancreatic cancers are already unresectable when they are initially diagnosed. Therefore, the development of angiogenesis inhibitors poses several challenges to the identification of the optimal study design, the criteria for eligibility of the patients, the modalities and schedule of administration, and the criteria for the evaluation of their biologic and therapeutic efficacy in pancreatic cancer patients. In any case, at present, the inhibition of angiogenesis represents one of the most promising novel therapeutic strategies to improve the management of pancreatic cancer.

### ACKNOWLEDGEMENTS

This work was supported in part by Grants-in-Aid from the Ministry of Education, Science and Culture of Japan to MM (07557253 and 08407040).

© Cancer Research Campaign 1999 

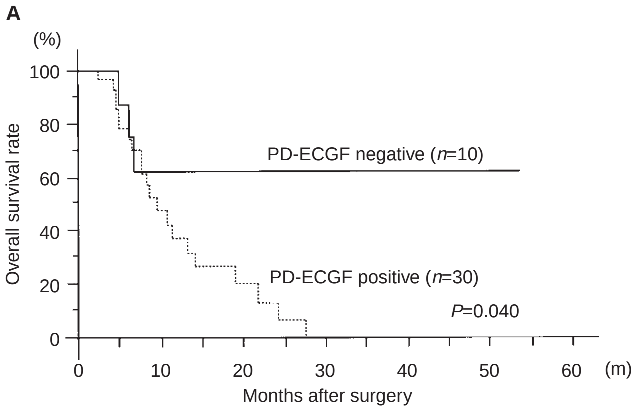

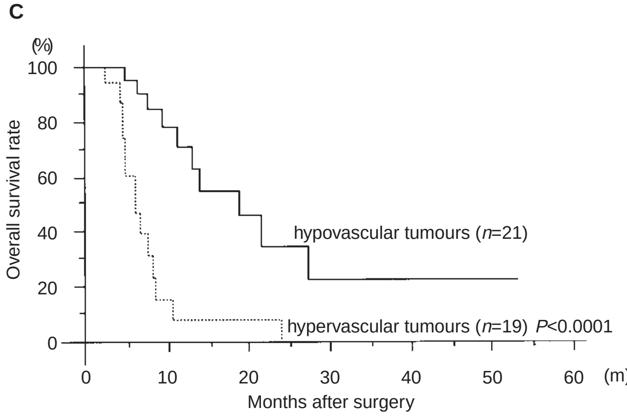

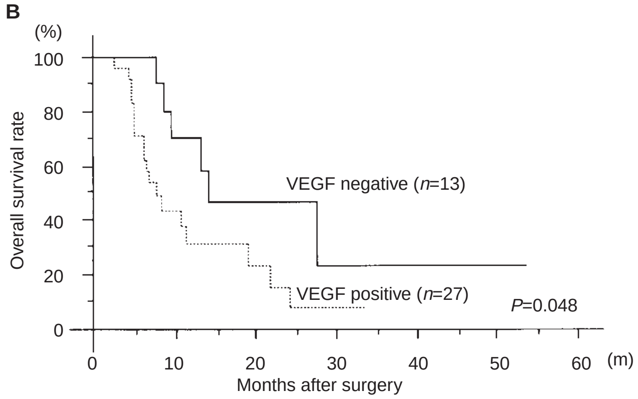

**Figure 5** Overall survival of 40 patients with pancreatic cancer in relation to *PD-ECGF* gene status (**A**). The median survival of 30 patients positive for *PD-ECGF* gene expression was 9.5 months, and the 1- and 3-year survival rates were 36.9%, and 0%, respectively. In contrast, The median survival of 10 patients negative for *PD-ECGF* gene expression was not reached, and the 1- and 3-year survival rates were 62.5% and 62.5%, respectively. Overall survival of 40 patients with pancreatic cancer in relation to *VEGF* gene status (**B**). The median survival of 27 patients positive for *VEGF* gene expression was 7.5 months, and the 1- and 3-year survival rates were 31.3% and 7.8%, respectively. In contrast, The median survival of patients negative for *VEGF* gene expression was 14 months, and the 1- and 3-year survival rates were 70.0% and 23.3%, respectively. Overall survival of 40 patients with pancreatic cancer in relation to IMD status (**C**). The median survival of 19 patients with hypervascular tumours was 6.3 months, and the 1- and 3-year survival rates were 7.8% and 0%, respectively. In contrast, the median survival of 21 patients negative for IMD status was 18.8 months, and the 1- and 3-year survival rates were 70.8% and 22.9%, respectively

### REFERENCES

Aiello LP, Avery RL, Arrigg PG, Keyt BA, Jampel HD, Shah ST, Pasquale LR, Thieme H, Iwamoto MA, Park JE, Nguyen HV, Aiello LM, Ferrara N and King GL (1994) Vascular endothelial growth factor in ocular fluid of patients with diabetic retinopathy and other retinal disorders. *N Engl J Med* **331**: 1480–1487

Barton CM, Hall PA, Hughes CM, Gullick WJ and Lemoine NR (1991) Transforming growth factor alpha and epidermal growth factor in human pancreatic cancer. *J Pathol* **163**: 111–116

Bramhall SR, Allum WH, Jones AG, Allwood A, Cummins C and Neoptolemos JP (1995) Treatment and survival in 13 560 patients with pancreatic cancer, and incidence of the disease, in the West Midlands: an epidemiological study. *Br J Surg* **82**: 111–115

Brogi E, Schatteman G, Wu T, Kim EA, Varticovski L, Keyt B and Isner JM (1996) Hypoxia-induced paracrine regulation of vascular endothelial growth factor receptor expression. *J Clin Invest* **97**: 469–476

Chomczynski P and Sacchi N (1987) Single-step method of RNA isolation by acid guanidinium thiocyanate–phenol–chloroform extraction. *Anal Biochem* **162**: 156–159

Cox DR (1972) Regression models and life-tables. *J R Stat Soc B* **34**: 187–220

Detmar M, Brown LF, Claffey KB, Yeo KT, Kocher O, Jackman RW, Berse B and Dvorak HF (1994) Overexpression of vascular permeability factor/vascular endothelial growth factor and its receptors in psoriasis. *J Exp Med* **180**: 1141–1146

Dvorak HF, Brown LF, Detmar M and Dvorak AM (1995) Vascular permeability factor/vascular endothelial growth factor, microvascular hyperpermeability, and angiogenesis. *Am J Pathol* **146**: 1029–1039

Fava RA, Olsen NJ, Spencer-Green G, Yeo KT, Yeo TK, Berse B, Jackman RW, Senger DR, Dvorak HF and Brown LF (1994) Vascular permeability factor/endothelial growth factor (VPF/VEGF): accumulation and expression in human synovial fluids and rheumatoid synovial tissue. *J Exp Med* **180**: 341–346

Fidler IJ and Ellis LM (1994) The implications of angiogenesis for the biology and therapy of cancer metastasis. *Cell* **79**: 185–188

Folkman J (1995*a*) Clinical applications of research on angiogenesis. *N Engl J Med* **333**: 1757–1763

Folkman J (1995*b*) Angiogenesis in cancer, vascular, rheumatoid and other disease. *Nature Med* **1**: 27–31

Friess H, Yamanaka Y, Büchler M, Ebert M, Beger HG, Gold LI and Korc M (1993) Enhanced expression of transforming growth factor β isoforms in pancreatic cancer correlates with decreased survival. *Gastroenterology* **105**: 1846–1856

Furukawa T, Yoshimura A, Sumizawa T, Haraguchi M and Akiyama S (1992) Angiogenic factor. *Nature (Lond.)* **356**: 668

Gastrointestinal Tumor Study Group (1987) Further evidence of effective adjuvant combined radiation and chemotherapy following curative resection of pancreatic cancer. *Cancer (Phila.)* **59**: 2006–2010

Hanahan D and Folkman J (1996) Patterns and emerging mechanisms of the angiogenic switch during tumorigenesis. *Cell* **86**: 353–364

Houck KA, Leung DW, Rowland AM, Winer J and Ferrara N (1992) Dual regulation of vascular endothelial growth factor bioavailability by genetic and proteolytic mechanisms. *J Biol Chem* **267**: 26031–26037

Ishikawa F, Miyazono K, Hellman U, Drexler H, Wernstedt C, Hagiwara K, Usuki K, Takaku F, Risau W and Heldin CH (1989) Identification of angiogenic activity and the cloning and expression of platelet-derived endothelial cell growth factor. *Nature (Lond.)* **338**: 557–562

Itakura J, Ishiwata T, Friess H, Fujii H, Matsumoto, Büchler MW and Korc M (1997) Enhanced expression of vascular endothelial growth factor in human pancreatic cancer correlates with local disease progression. *Clin Cancer Res* **3**: 1309–1316

Kamthan AG, Morris JC, Dalton J, Mandeli JP, Chesser MR, Leben D, Cooperman A and Bruckner HW (1997) Combined modality therapy for stage II and stage III pancreatic carcinoma. *J Clin Oncol* **15**: 2920–2927

Kaplan EL and Meier P (1958) Nonparametric estimation from incomplete observations. *J Am Stat Assoc* **53**: 457–481

Kim KJ, Li B, Winer J, Armanini M, Gillett N, Phillips HS and Ferrara N (1993) Inhibition of vascular endothelial growth factor-induced angiogenesis suppresses tumor growth in vivo. *Nature* **362**: 841–844

Mantel N (1966) Evaluation of survival data and two new rank order statistics arising in its consideration. *Cancer Chemother Rep* **50**: 163–170

McCulloch P, Choy A and Martin L (1995) Association between tumor angiogenesis and tumor cell shedding into effluent venous blood during breast cancer surgery. *Lancet* **346**: 1334–1335

*© Cancer Research Campaign 1999*

Millauer B, Shawver LK, Plate KH, Risau W and Ullrich A (1994) Glioblastoma growth inhibited in vivo by a dominant-negative Flk-1 mutant. *Nature* **367**: 576–579

Minari Y, Nio Y, Sato Y, Omori H, Takeda H, Hirahara N, Sumi S and Tamura K (1997) The clinicopathological significance of thymidine phosphorylase in human pancreatic cancer: Its different distribution between the primary and the metastatic lesions. *J Jpn Soc Cancer Ther* **32**: 315–325

Nakajima-Iijima S, Hamada H, Reddy P and Kakunaga T (1985) Molecular structure of the human cytoplasmic β-actin gene: interspecies homology of sequences in the introns. *Proc Natl Acad Sci USA* **82**: 6133–6137

Nitecki SS, Michael GS, Colby TV and van Heerden JA (1995) Long-term survival after resection for ductal adenocarcinoma of the pancreas. Is it really improving? *Ann Surg* **221**: 59–66

Ozaki H, Kinoshita T, Kosuge T, Yamamoto J, Shimada K, Inoue K, Koyama Y and Mukai K (1996) An aggressive therapeutic approach to carcinoma of the body and tail of the pancreas. *Cancer* **77**: 2240–2245

Rak J, Mitsuhashi Y, Bayko L, Filmus J, Shirasawa S, Sasazuki T and Kerbel RS (1995) Mutant ras oncogenes upregulate VEGF/VPF expression: implications for induction and inhibition of tumor angiogenesis. *Cancer Res* **55**: 4575–4580

Ramakrishnan S, Olson TA, Bautch VL and Mohanraj D (1996) Vascular endothelial growth factor-toxin conjugate specifically inhibits KDR/flk-1-positive endothelial cell proliferation in vitro and angiogenesis in vivo. *Cancer Res* **56**: 1324–1330

Schnall SF and Macdonald JS (1996) Chemotherapy of adenocarcinoma of the pancreas. *Seminars in Oncol* **23**: 220–228

Shimoyama S, Gansauge F, Gansauge S, Negri G, Oohara T and Beger HG (1996) Increased angiogenin expression in pancreatic cancer is related to cancer aggressiveness. *Cancer Res* **56**: 2703–2706

Sobin LH and Wittekind CH (1997) TNM classification of malignant tumors. In *International Union Against Cancer (UICC)*, 5th ed. pp 87–90. John Wiley New York

Takahashi Y, Bucana CD, Liu W, Yoneda J, Kitadai Y, Cleary KR and Ellis LM (1996) Platelet-derived endothelial cell growth factor in human colon cancer angiogenesis: Role of infiltrating cells. *J Natl Cancer Inst* **88**: 1146–1151

Takahashi Y, Bucana CD, Akagi Y, Liu W, Cleary KR, Mai M and Ellis LM (1998) Significance of platelet-derived endothelial cell growth factor in the angiogenesis of human gastric cancer. *Clin Cancer Res* **4**: 429–434

Takebayashi Y, Akiyama S, Akiba S, Yamada K, Miyadera K, Sumizawa T, Yamada Y, Murata F and Aikou T (1996) Clinicopathologic and prognostic significance of an angiogenic factor, thymidine phosphorylase, in human colorectal carcinoma. *J Natl Cancer Inst* **88**: 1110–1117

Tandon AK, Clark GM, Chamness GC, Chirgwin JM and McGuire WL (1990) Cathepsin D and prognosis in breast cancer. *N Engl J Med* **322**: 297–302

Tischer E, Mitchell R, Hartman T, Silva M, Gospodarowicz D, Fiddes JC and Abraham JA (1991) The human gene for vascular endothelial growth factor. Multiple protein forms are encoded through alternative exon splicing. *J Biol Chem* **266**: 11947–11954

Tsurumi Y, Murohara T, Krasinski K, Chen D, Witzenbichler B, Kearney M, Couffinhal T and Isner JM (1997) Reciprocal relation between VEGF and NO in the regulation of endothelial integrity. *Nature Med* **3**: 879–886

Usuki K, Saras J and Waltenberger J (1992) Platelet-derived endothelial cell growth factor has thymidine phosphorylase activity. *Biochem Biophys Res Commun* **184**: 1311–1316

Weidner N and Folkman J (1996) Tumoral vascularity as a prognostic factor in cancer. In *Important Advances in Oncology 1996*, DeVita VT Jr, Hellman S and Rosenberg SA (eds) pp 167–190. Lippincott-Raven: Philadelphia

Yamamoto Y, Toi M, Kondo S, Matsumoto T, Suzuki H, Kitamura M, Tsuruta K, Taniguchi T, Okamoto A, Mori T, Yoshida M, Ikeda T and Tominaga T (1996) Concentrations of vascular endothelial growth factor in the sera of normal controls and cancer patients. *Clin Cancer Res* **2**: 821–826

Yamanaka Y, Friess H, Buchler M, Beger HG, Uchida E, Onda M, Kobrin MS and Korc M (1993) Overexpression of acidic and basic fibroblast growth factors in human pancreatic cancer correlates with advanced tumor stage. *Cancer Res* **53**: 5289–5296

Yoshiji H, Gomez DE, Shibuya M and Thorgeirsson UP (1996) Expression of vascular endothelial growth factor, its receptor, and other angiogenic factors in human breast cancer. *Cancer Res* **56**: 2013–2016

© Cancer Research Campaign 1999
